# Electrochemical Performances of Electroactive Nano-Layered Organic-Inorganic Perovskite Containing Trivalent Iron Ion and its Use for a DNA Biosensor Preparation

**DOI:** 10.1155/2010/419439

**Published:** 2010-06-29

**Authors:** Jing Wu, Hanxing Liu, Zhidong Lin, Minghe Cao

**Affiliations:** ^1^State Key Laboratory of Advanced Technology for Materials Synthesis and Processing, Wuhan University of Technology, Wuhan 430070, China; ^2^State-key Laboratory of Chemo/Biosensing and Chemometrics, College of Chemistry and Chemical Engineering, Hunan university, Changsha, Hunan 410082, China; ^3^School of Materials Science Engineering, Wuhan Institute of Technology, Wuhan, Hubei 430073, China

## Abstract

A steady nano organic-inorganic perovskite hybrid with [H_2_3-AMP]_3/2_Fe(CN)_6_ (3-AMP = 3-methylaminopyridine) was prepared in the air. The structure is an unusual layered organic-inorganic type. The resulting hybrid enveloped in paraffin to prepare [H_2_3-AMP]_3/2_Fe(CN)_6_ paste electrode (HPE) shows good electrochemical activity and a couple of oxidation and reduction peaks with potential of cyclic voltammometry (CV) at around 440 mV and 30 mV. Compared with that on CPE, oxidation potential of Fe(CN)_6_^3−^ on HPE shifts negatively 259.7 mV and that of reduction shifts positively 338.7 mV, which exhibits that [H_2_3-AMP]_3/2_Fe(CN)_6_ can accelerate the electron-transfer to improve the electrochemical reaction reversibility. Such characteristics of [H_2_3-AMP]_3/2_Fe(CN)_6_ have been employed to prepare the DNA biosensor. The single-strand DNA (ssDNA) and double-strand DNA (dsDNA) immobilized on HPE, respectively, can improve the square wave voltammometry (SWV) current and SWV potential shifts positively. The effect of pH was evaluated. And there is hybridization peak on SWV curve using HPE immobilized ssDNA in the complementary ssDNA solution. And HPE immobilized ssDNA can be utilized to monitor the DNA hybridization and detect complementary ssDNA, covering range from 3.24 × 10^−7^ to 6.72 × 10^−5^ g/mL with detection limit of 1.57 × 10^−7^ g/mL. The DNA biosensor exhibits a good stability and reproducibility.

## 1. Introduction

 In recent years, layered organic-inorganic perovskite hybrids have attracted great interest of researchers. Organic-inorganic perovskite hybrids offer an important opportunity to combine useful properties from two chemical realms, organic and inorganic compounds, within a single molecular scale composite. Especially, the design and generation of the organic-inorganic self-organized quantum well structure represent an approach to synthesis that offers new horizons in the context of synthetic chemistry and its possible impact on new material technology [1].

 The hybrid organoammonium halometallate(II), (RNH_3_)_2_MX_4_, series of layer perovskite structures have played a significant role in the development of the understanding of low-dimensional magnetic systems and, more recently, of semiconducting, optical, photoelectric materials [2–6]. So far, only organic-inorganic perovskite hybrids containing the chemical element of tin (Sn), copper (Cu), and lead (Pb) have been used to application [7–13]. While it is not reported that organic-inorganic perovskite hybrids were used as electrochemical materials.

On the other hand, a few organic-inorganic perovskite hybrids containing M^3+^ have been reported. As for conventional perovskite frameworks, trivalent metal ions halide lattices generally are composed of distorted MX_6_ (X = halogen) octahedra. These MX_6_ octahedra form discrete (i.e., mononuclear) or extended (i.e., polynuclear) inorganic networks of corner-, edge-, or face-sharing octahedra, leading to an extensive family of metal (III) halogenoanions (e.g., MX_4_^−^, MX_5_^2−^, MX_6_^3−^, M_2_X_9_^3−^, M_2_X_11_^5−^, M_3_X_12_^3−^, M_4_X_18_^6−^, M_5_X_18_^3−^, M_6_X_22_^4−^, and M_8_X_30_^6−^). Within these networks, the inorganic sections of perovskite hybrids incline to diversification. Mitzi's group has successfully synthesized thin sheetlike crystals of the metal-deficient perovskites (H_2_AEQT)M_2/3_I_4_ [M = Bi^3+^ or Sb^3+;^ AEQT = 5,5-bis-(aminoethyl)-2,2:5,2:5,2-quaterthiophene] under an inert atmosphere [14]. The stability of (H_2_AEQT)  M_2/3_I_4_ needs to be improved. In this study, we utilized the Fe(CN)_6_^3−^ as inorganic sheets of perovskite hybrids to prepare steady layered organic-inorganic perovskite hybrids containing M^3+^, which to avoid the an extensive family of metal (III) halogenoanions.

In present work, we prepared layered organic-inorganic perovskite hybrid—[H_2_3-AMP]_3/2_Fe(CN)_6_ by molecular scale steric interaction between 3-(methylAmino)pyridine and Fe(CN)_6_^3−^. And the microstructure of [H_2_3-AMP]_3/2_· Fe(CN)_6_ was characterized. [H_2_3-AMP]_3/2_Fe(CN)_6_ was utilized to electrochemical material. The electrochemical redox and electrocatalytic characteristics of [H_2_3-AMP]_3/2_Fe(CN)_6_ were investigated by [H_2_3-AMP]_3/2_Fe(CN)_6_ paste electrode (HPE). DNA biosensor based on HPE which can detect the hybridization process was explored. The interaction between DNA and [H_2_3-AMP]_3/2_Fe(CN)_6_, the hybridization process, pH effect and linear range will be investigated in the article.

## 2. Experimental Section

### 2.1. Synthesis

Organic-inorganic perovskite hybrids in this study were prepared in 4 mol·L^−1^ aqueous hydrochloric acid solution in the air. Organic-inorganic perovskite hybrids were prepared by reacting potassium Ferricyanide and amine in hydrochloric acid. The ratio of AMP and potassium Ferricyanide is 3 : 2. For the [H_2_3-AMP]_3/2_Fe(CN)_6_ system, a total weight of 0.491 g of 2-AMP (0.162 g 1.5 mmol) and K_3_Fe(CN)_6_ (0.329 g, 1 mmol) were dissolved in 20 mL of 4 mol·L^−1^ aqueous hydrochloric acid solution. Heating was in a water bath at 65°C for 1 hour to form deposition. The hybrid products were filtered from which sheet-like crystals were obtained. The products were dried in vacuum drying box at 65°C for 12 hours and then removed to a dry box with the water levels maintained below 50 ppm. IR data: *ν* (NH_3_^t^): 3212, 3041, 2952; *δ* (NH_3_^t^): 1716, 1606; *ν* (pyridinium): 1571, 1573, 1550, 1507; *δ* (Ar-H): 822, 787, 688; *ν* (-CH_2_-): 2932, 2893, 2853; *ν* (CN^−^):2079, 2166. These TR data are similar to those in [15], which indicate that the resulting hybrid is pure. Elemental analysis: calculated for (H_2_3-AMP)_3/2_Fe(CN)_6_: C, 48.01%; H, 3.98%; N, 33.42%. Found: C, 48.61%; H, 4.12%; N, 33.28%.

### 2.2. Characterization

 FT-IR spectra were measured with JASCO FT/IR-4000 infrared spectrophotometer with the KBr pellet technique. Elemental analyses were carried out on Vario EL instrument. The X-ray diffraction (XRD) of the powder samples was examined on a Rigaku D/Max-IIIA X-ray Diffractometer (XRD) using Cu Ka radiation. All cyclic voltammograms data were acquired using a computer-based potentiostat/galvanostat (model 283) (EG&GP Princeton Applied Research, Princeton, NJ, USA). The three-electrode system consists of a sensing device, an Ag/AgCl reference electrode, and a platinum wire as an auxiliary electrode.

### 2.3. Preparation of the HPE

HPE was used as the matrix electrode. It was prepared according to the procedure reported elsewhere [16] with minor modifications. In a typical process, an appropriate amount (79.9 mg) of [H_2_3-AMP]_3/2_Fe(CN)_6_ was mixed with 150.0 mg of graphite powder and left to oscillate in ultrasonic vibrator for 2 h to form a homogeneous mixture. Paraffin (149.6 mg) was dissolved in ether and dried in air about 10 min. After evaporation of the excess of ether, the paraffin paste is mixed with the homogeneous mixture. The resulting paste was squeezed into the designed device to a depth of 1 cm. Inside the tube, the mass was in contact with a conducting graphite rod, which was in turn connected with an electric wire to complete the measurement circuit.

### 2.4. Immobilization of ssDNA and dsDNA on HPE

HPE surface was first polished on a piece of emery paper, smoothed again on weighing paper, and rinsed with water. Subsequently, the cleaned HPE was immersed in 1 mL of tris buffer containing 50 *μ*L of 1.57 mg/mL calf thymus ssDNA at 4°C for 4 hours, rinsed with water. The modified electrode was then put at the same temperature to dry. Single-strand DNA was immobilized on the HPE.

The process of immobilization of calf thymus dsDNA on HPE is similar to that of immobilization of ssDNA only with ssDNA replaced with dsDNA.

### 2.5. Measurement Procedure

 SWV measurements were performed in 10 mL of tris buffer of pH 7.2. A three-electrode system was used with a potential range from 2.0 mV to −1000 mV. When the background was stabilized, different volumes of solutions of 1.57 mg/mL ssDNA were added, the signal was recorded.

## 3. Results and Discussion

### 3.1. Characterization of [H_2_3-AMP]_3/2_Fe(CN)_6_

**Figure 1:**
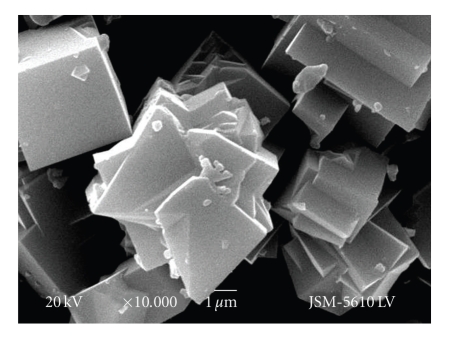
SEM image of [H_2_3-AMP]_3/2_Fe(CN)_6_.

[H_2_3-AMP]_3/2_Fe(CN)_6_ hybrids crystallize in a monoclinic crystal system with space group *P*121 (3). The structure adopts an unusual layered organic-inorganic chain-like structure with the cell parameters *a* = 23.79(2) Å, *b* = 11.49(1) Å, *c* = 6.77(1) Å, *β* = 106.2(6)°, *α* = *γ* = 90°, *Z* = 8, and Volume = 1775.04. Figure 1 shows the microstructure of (H_2_3-AMP)_3/2_Fe(CN)_6_. The [H_2_3-AMP]_3/2_Fe(CN)_6_ sample is composed of some regularly sandwich stacking with different thickness and each layer is around 100–200 nm. One can see that each layer cross-section shows layered distribution, which is attributed to alternate arrangement between organic sheets and inorganic ones in the perovskite hybrids. It is well known that CN^−^ in Fe(CN)_6_^3−^ have bridge-linked action to make organic-inorganic perovkite hybrids containing trivalent iron ions stable.

**Figure 2:**
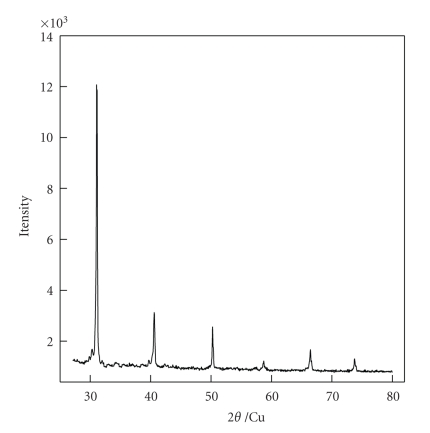
X-ray diffraction patterns showing the reflections of [H_2_3-AMP]_3/2_Fe(CN)_6_.

The X-ray diffraction (XRD) pattern of the sample is shown in Figure 2. The compound exhibits well-defined and equally spaced diffraction peaks. Results of the XRD study indicate that the sample—[H_2_3-AMP]_3/2_Fe(CN)_6_—is well crystallized and highly oriented and has a typical layered structure. The result is consistent with that of SEM.

### 3.2. Electrochemical Activity of [H_2_3-AMP]_3/2_Fe(CN)_6_

**Figure 3:**
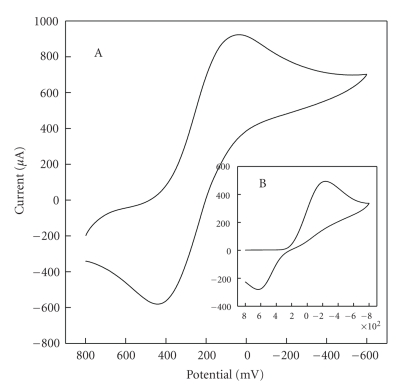
Cyclic voltammograms of HPE in 0.1 mol·L^−1^ KCl solution of pH 7.0 (A) and CPE 0.01 mol·L^−1^  Fe(CN)_6_^3−^ containing 0.1 mol·L^−1^ KCl solution of pH 7.0 (B). scan rate: 50 mV·s^−1^.

Figure 3(A) shows the cyclic voltammometry (CV) curves of HPE 50 mV/s in 0.1 ml·L^−1^ KCl solution of pH 7.0. One notices that the curve of HPE based on [H_2_3-AMP]_3/2_Fe(CN)_6_  reveals one couple of redox peaks under the potentials of around 440 mV and 30 mV, respectively. Is the oxidation and reduction of HPE from Fe(CN)_6_^3−^, AMP, or organic-inorganic perovskite hybrids? Compared with that of Fe(CN)_6_^3−^ on carbon paste electrode (CPE) (Figure 3(B)), the oxidation peak potential shifts negatively at 193 mV and reduction potential shifts positively at 267 mV. On the other hand, it is reported that the oxidation potential of AMP is more than 1000 mV [17]. All results demonstrate that redox process is attributed to the quasireversible redox of [H_2_3-AMP]_3/2_Fe(CN)_6_ and redox activity of [H_2_3-AMP]_3/2_Fe(CN)_6_ is satisfactory. 

The relationship between peak current and potential scan rate can embody the redox mechanism. The influence of potential scan rate on the oxidation and reduction peak current has been investigated by cyclic voltammetry using HPE. It can be observed that the redox peak current of HPE is proportional to the root of scan rate, which indicates that the current is controlled by a semiinfinite linear diffusion. The main reason of these experimental results is that the redox chemicals [H_2_3-AMP]_3/2_Fe(CN)_6_ are on the surface of HPE. On the other hand, the redox peak potential shifts slightly with the scan rate increase, which indicates that the physical and chemical properties of [H_2_3-AMP]_3/2_Fe(CN)_6_ are stable.

On the basis of the above results, the redox mechanism of [H_2_3-AMP]_3/2_Fe(CN)_6_ may be expressed as follows. The molecule of 3-AMP possesses the rich electron structure and easily forms *π*-conjugated structure, which is favorable to electron-transfer during reduction and oxidation process and promotes the [H_2_3-AMP]_3/2_Fe(CN)_6_  redox behavior. On the other hand, the inorganic layers are consisted of Fe(CN)_6_^3−^ which is a typical electrochemical probe and have good reduction and oxidation characteristics. Combined with the Fe(CN)_6_^3−^ inorganic layers and 3-AMP organic layers, the electrochemical characteristics of [H_2_3-AMP]_3/2_Fe(CN)_6_ are more active than that of Fe(CN)_6_^3−^ and 3-AMP. 

**Figure 4:**
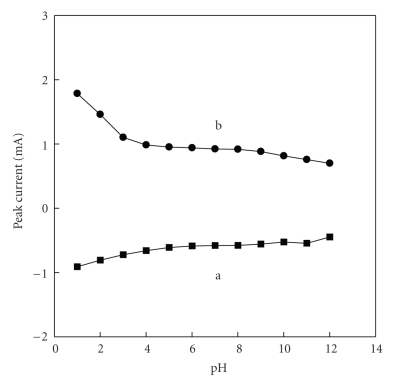
Effect of pH and the redox peak current of [H_2_3-AMP]_3/2_Fe(CN)_6_ (a) oxidization (b) reduction.

Figure 4 shows that the redox peak current of [H_2_3-AMP]_3/2_Fe(CN)_6_ on electrode can be affected by the value of pH. The redox peak current decreases with the increase of the value of pH, covering linear range from 1.0 to 6.0. And it is observed that the response current levels off significantly from pH 6.0 to pH 8.0. Above pH 8.0, the peak current tends to directly decrease with the increase of pH. Additionally, the redox potential shifts slightly with the change of pH. The experimental results show that the reduction and oxidation of [H_2_3-AMP]_3/2_Fe(CN)_6_ are more active in the acid solution than that in the alkaline solution. This may be attributed to the resulting hybrid which was prepared in the acid solution.

### 3.3. Electrochemical Catalysis Characteristics of [H_2_3-AMP]_3/2_Fe(CN)_6_

**Figure 5:**
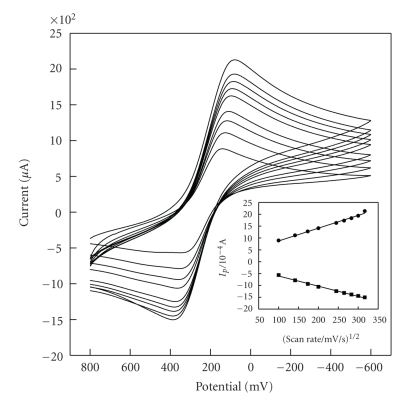
Cyclic voltammograms of HPE in 0.01 mol·L^−1^  Fe(CN)_6_^3−^ containing 0.1 mol·L^−1^ KCl solution of pH 7.0. at various scan rates (from inner to outer curves: 10, 20, 30, 40, 60, 70, 80, 90, and 100 mV·s^−1^).

The HPE was used to investigate the electrochemistry of Fe(CN)_6_^3−^. Using potassium ferricyanide cyclic voltammetric current recorded as a function of scan rate shows a linear *I*_*p*_ versus v^1/2^ relationship covering the 10–100 mV/s range, as shown in Figure 5. This indicates that the current is controlled by a semiinfinite linear diffusion. The redox potential of Fe(CN)_6_^3−^ on HPE shifts slightly with the change of scan rate. The results indicate that catalysis action of [H_2_3-AMP]_3/2_Fe(CN)_6_ is very nice and stable.

**Figure 6:**
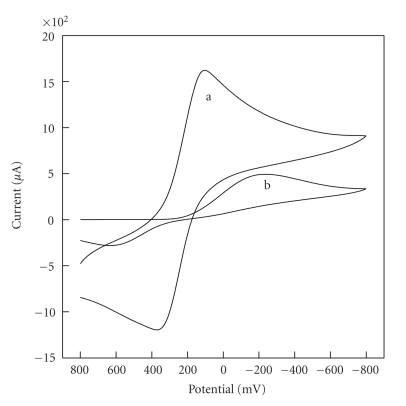
Cyclic voltammograms of HPE (a) and CPE (b) in 0.01 mol·L^−1^  Fe(CN)_6_^3−^ containing 0.1 mol·L^−1^ KCl solution of pH 7.0. Scan rate: 50 mV·s^−1^.

The electrochemical characteristics of HPE were studied by cyclic voltammetry in 0.1 ml·L^−1^ KCl solution of pH 7.0 and 20 mM potassium ferricyanide solution at a scan rate of 50 mV/s. The advantages of these new electrodes were compared with conventional CPE, as shown in Figure 6. Fe(CN)_6_^3−^ (Figure 6(a)) on HPE displays a couple of redox peaks with peak potential 101.3 and 373.3 mV, respectively. The oxidation and reduction potential shifts negatively 259.7 mV–338.7 mV, respectively, compared with that on CPE (Figure 6(b)). For reduction and oxidation of Fe(CN)_6_^3−^, a high degree reversible reaction was observed at HPE (Δ*E*_*p*_ = 272.0 mV). In fact, the electrochemical oxidation of Fe(CN)_6_^3−^ was investigated at different concentrations, ranging from 0.001 mM to 10 mM, at HPE. There is a linear relationship between the Fe(CN)_6_^3−^ concentration and oxidation peak current with *γ* = 0.9998. All experimental results indicate that HPE can accelerate the electron-transfer to improve the electrochemical reaction reversibility, as compared with the conventional CPE. That is to say, [H_2_3-AMP]_3/2_Fe(CN)_6_ is a good kind of electrochemical catalyst.

### 3.4. Stability of [H_2_3-AMP]_3/2_Fe(CN)_6_

**Figure 7:**
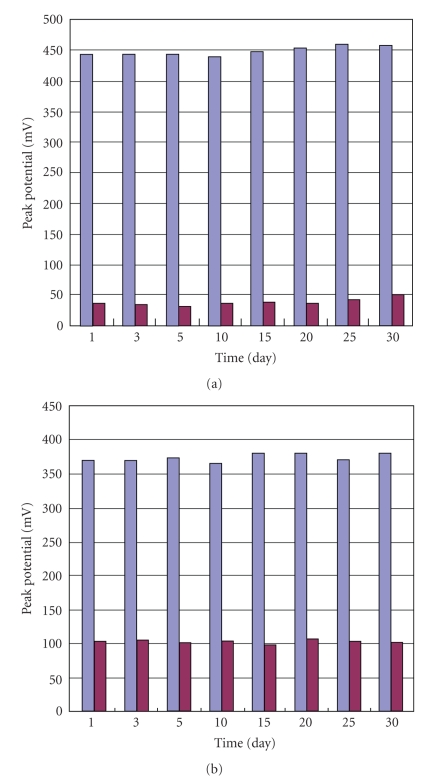
The relationship between redox potential and time in 0.1 mol·L^−1^ KCl (a) and 0.01 mol·L^−1^  Fe(CN)_6_^3−^ containing 0.1 mol·L^−1^ KCl (b) solution of pH 7.0.

The above electrochemical experiments have demonstrated that [H_2_3-AMP]_3/2_Fe(CN)_6_ has good chemical and physical stability. In order to further investigate their stability, the stability of response current of HPE was carried out in 0.01 mol·L^−1^  Fe(CN)_6_^3−^ and 0.1 ml·L^−1^ KCl solution by cyclic voltammetry. The variation coefficients (RSD) of HPE are 1.3% and 1.9%, respectively, for five successive assays. For five interval assays, RSDs are 1.9% and 2.3%, respectively. Furthermore, HPE kept in desiccator with the air for more than a month, while the redox potential changes slightly, as shown in Figure 7. The results show that HPE has a promising stability and reproducibility. From another point of view, the results demonstrate that [H_2_3-AMP]_3/2_Fe(CN)_6_ has a finer electrochemical stability.

### 3.5. DNA Biosensor Based on [H_2_3-AMP]_3/2_Fe(CN)_6_

**Figure 8:**
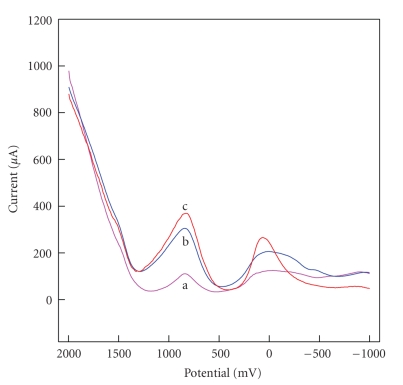
SWV curves using HPE (a), HPE immobilized with ssDNA (b), and HPE immobilized with dsDNA (c) in tris buffer solution.

The square wave voltammograms of HPE and HPE immobilized with ssDNA and dsDNA in tris buffer solution are shown in Figure 8. It is obvious that two SWV peaks of [H_2_3-AMP]_3/2_Fe(CN)_6_ at 840.0 mV and −22.8 mV (Figure 8(a)) are present. On HPE-immobilized ssDNA, the peak of low potential shifts positively and the other one shifts little, as shown in Figure 8(b). When HPE was immobilized with dsDNA, the two SWV peaks (863.5 and 64.0 mV, resp.) shift positively. The results demonstrate that ssDNA and dsDNA can change redox characteristics of [H_2_3-AMP]_3/2_Fe(CN)_6_ and the [H_2_3-AMP]_3/2_Fe(CN)_6_ has a different electrocatalytic effect on ssDNA and dsDNA.

**Figure 9:**
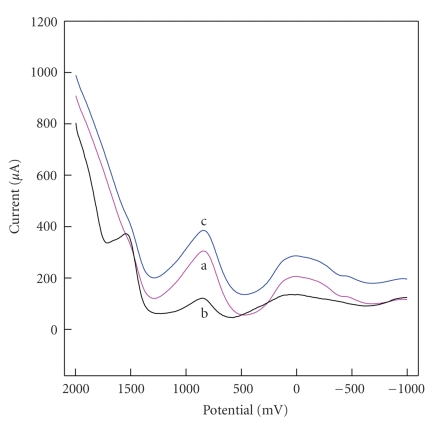
SWV curves using HPE immobilized with ssDNA in tris buffer solution (a) with the complementary ssDNA (b) and ssDNA of fish semen.

 Figures 9(a) and 9(b) show the SWVs recorded on an ssDNA-immobilized HPE in tris buffer solution with and without the complementary ssDNA. Two peaks of ssDNA-immobilized HPE (Figure 9(a)) are observed in solution without the complementary ssDNA, which is similar to that of Figure 8(b). When the electrode immobilized with ssDNA is put in the solution containing the complementary ssDNA and the SWV scan is performed again, two primary peak current decreases dramatically and a new SWV peak appears at 1.544 mV, as shown in Figure 9(b). It is possible that the new redox current is the signal of hybridization between ssDNA on HPE and the complementary ssDNA in solution. In order to further confirm the new peak being the signal of DNA hybridization, the calf thymus ssDNA-immobilized electrode is scanned in the solution containing ssDNA of fish semen, as shown in Figure 9(c). One can see that there is no peak at 1.544 mV; only the redox current of ssDNA is observed. These results show that the new peak is really a signal of hybridization, that is, HPE immobilized ssDNA can be utilized to monitor the DNA hybridization and [H_2_3-AMP]_3/2_Fe(CN)_6_ can be used to prepare the DNA biosensor.

According to the above results, the interaction between [H_2_3-AMP]_3/2_Fe(CN)_6_ and DNA is thought to take place in the following way. Because [H_2_3-AMP]_3/2_Fe(CN)_6_ has rich NH_3_^+^ and Fe(CN)_6_ and has unusual layered organic-inorganic structural type, it may interact with ssDNA and dsDNA through ordinary interaction mechanism, that is, electrostatic interaction and hydrogen bonding, together with layered structure interactions. And ssDNA and dsDNA immobilized on HPE can change redox characteristics of (H_2_3-AMP)_3/2_Fe(CN)_6_. And interaction between ssDNA and [H_2_3-AMP]_3/2_Fe(CN)_6_ is different from that between dsDNA and [H_2_3-AMP]_3/2_Fe(CN)_6_ which may be attributed to the different structure between ssDNA and dsDNA. When ssDNA on HPE hybridizes with the complementary ssDNA in solution to form dsDNA, there exists an obvious change on HPE surface to affect reduction and oxidation of [H_2_3-AMP]_3/2_Fe(CN)_6_. And layered structure of [H_2_3-AMP]_3/2_Fe(CN)_6_ is in favor of monitoring DNA of the hybridization [18], so [H_2_3-AMP]_3/2_Fe(CN)_6_ can detect the hybridization of DNA.

### 3.6. Effect of pH

**Figure 10:**
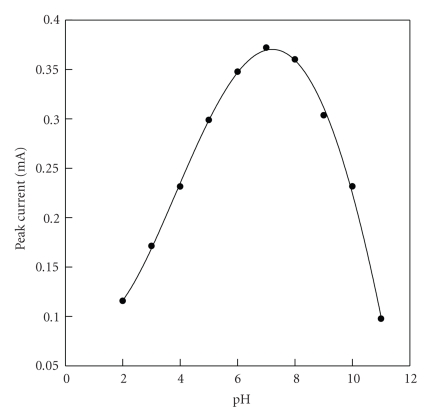
The relationship between the DNA hybridization peak current and pH.

The relationship between DNA hybridization peak current and pH autoxidant time from 2 to 11 was monitored as shown in Figure 10. It is observed that the response current increases significantly from pH 2 to pH 6. Above pH 8, the peak current tends to directly decrease with the increase of pH. The DNA hybridization has maximum sensitivity around pH 7. Combined with the results of Figure 9, pH 7 is selected for the determination of the complementary ssDNA in solution.

### 3.7. The Response Characteristics of HPE Immobilized with DNA

The repeatability of response current of HPE immobilized with ssDNA and dsDNA, respectively, was investigated in tris buffer solution. The variation coefficient (RSD) was 3.9% and 3.7%, respectively, for five successive assays. In the complementary ssDNA solution, HPE immobilized with ssDNA showed an acceptable reproducibility with a variation coefficient of 4.1%. The results show that the DNA biosensor based on [H_2_3-AMP]_3/2_Fe(CN)_6_ has a good repeatability and reproducibility.

**Figure 11:**
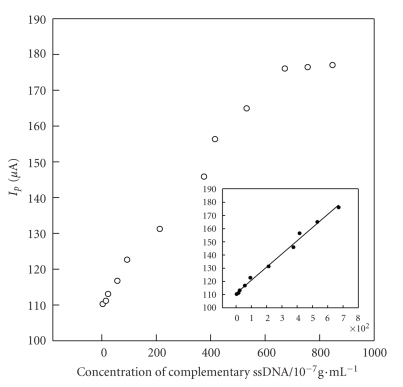
The complementary ssDNA determination using a DNA biosensor based on [H_2_3-AMP]_3/2_Fe(CN)_6_.

The SWV peak current was investigated at the HPE mobilized with ssDNA using different concentrations of the complementary ssDNA. With the concentrations increasing, the peak potential appears to shift slightly. The peak current increases with the increase of the complementary ssDNA concentration. Figure 11 displays the calibration curves for the complementary ssDNA using HPE immobilized with ssDNA. A linear relationship between the peak current and the complementary ssDNA concentration in solution was obtained covering range from 3.24 × 10^−7^ to 6.72 × 10^−5^ g/mL. The linear regression equation is *I*  (*μ* *A*) = 0.0998*C*  (10^−7^ g/mL) + 110.8, with a correlation coefficient (*γ*) of 0.9932. The detection limit is 1.57 × 10^−7^ g/mL. These indicate that the distribution of [H_2_3-AMP]_3/2_Fe(CN)_6_  in HPE is uniform and HPE immobilized with ssDNA provides reliable results in determination of ssDNA.

## 4. Conclusion

 The present study investigated the microstructure and electrochemical activity of organic-inorganic perovskite hybrid—[H_2_3-AMP]_3/2_Fe(CN)_6_ in detail. By molecular scale steric interaction between 3-(methylAmino)pyridine and ferricyanide, the formation of [H_2_3-AMP]_3/2_Fe(CN)_6_  is typical structure of layered organic-inorganic perovskite. [H_2_3-AMP]_3/2_Fe(CN)_6_ in HPE exhibits nice electrochemical redox and electrocatalytic characteristics and the electrochemical stability is satisfactory. The paraffin-[H_2_3-AMP]_3/2_Fe(CN)_6_ electrode (HPE) described in this paper provides a sensitive tool for monitoring the DNA hybridization process and detection of the complementary ssDNA. A wide linear range with *γ* = 0.9932 was obtained. The physical and chemical characteristics of HPE including mechanical stability and reproducibility of the results have been examined.
